# New insights into fibrotic signaling in hepatocellular carcinoma

**DOI:** 10.3389/fonc.2023.1196298

**Published:** 2023-11-22

**Authors:** Liang Shan, Fengling Wang, Weiju Xue, Dandan Zhai, Jianjun Liu, Xiongwen Lv

**Affiliations:** ^1^ Department of Pharmacy, The Second People’s Hospital of Hefei, Hefei Hospital Affiliated to Anhui Medical University, Hefei, Anhui, China; ^2^ Anhui Province Key Laboratory of Major Autoimmune Diseases, Anhui Medical University, Hefei, China; ^3^ Inflammation and Immune Mediated Diseases Laboratory of Anhui Province, Hefei, China; ^4^ The Key Laboratory of Major Autoimmune Diseases, Hefei, Anhui, China

**Keywords:** fibrotic signaling, hepatocellular carcinoma, hepatic fibrosis, hepatic stellate cell, hepatocellular carcinoma cell

## Abstract

Hepatocellular carcinoma (HCC) mostly occurs in the background of liver fibrosis, and activated hepatic stellate cells (HSCs) exist in HCC tissues and adjacent tissues. HSC activation is involved throughout the development of HCC precancerous lesions, which has gradually attracted the attention of related researchers. In addition, HCC can promote the activation of HSCs, which in turn accelerates the occurrence and development of HCC by promoting tumor angiogenesis. In this review, we reviewed 264 studies from PubMed and ScienceDirect to summarize and analyze current significant fibrotic signaling in HCC. As a result, we found 10 fibrotic signaling pathways that are closely related to the activation, proliferation, invasion, migration, and promotion of apoptosis of HCC cells. In addition, we found that crosstalk between various fibrotic signaling pathways of HCC, hypoxia-induced energy metabolic reprogramming of HCC cells, matrix stiffness and stemness of HCC cells, and ferroptosis of HCC cells and HSCs are the latest research hotspots. Furthermore, related drugs that have been found to target these 10 fibrotic signaling pathways of HCC are listed. Our study provides a new reference for developing anti-HCC drugs.

## 1 Introduction

### 1.1 Hepatocellular carcinoma

**Figure 1:**
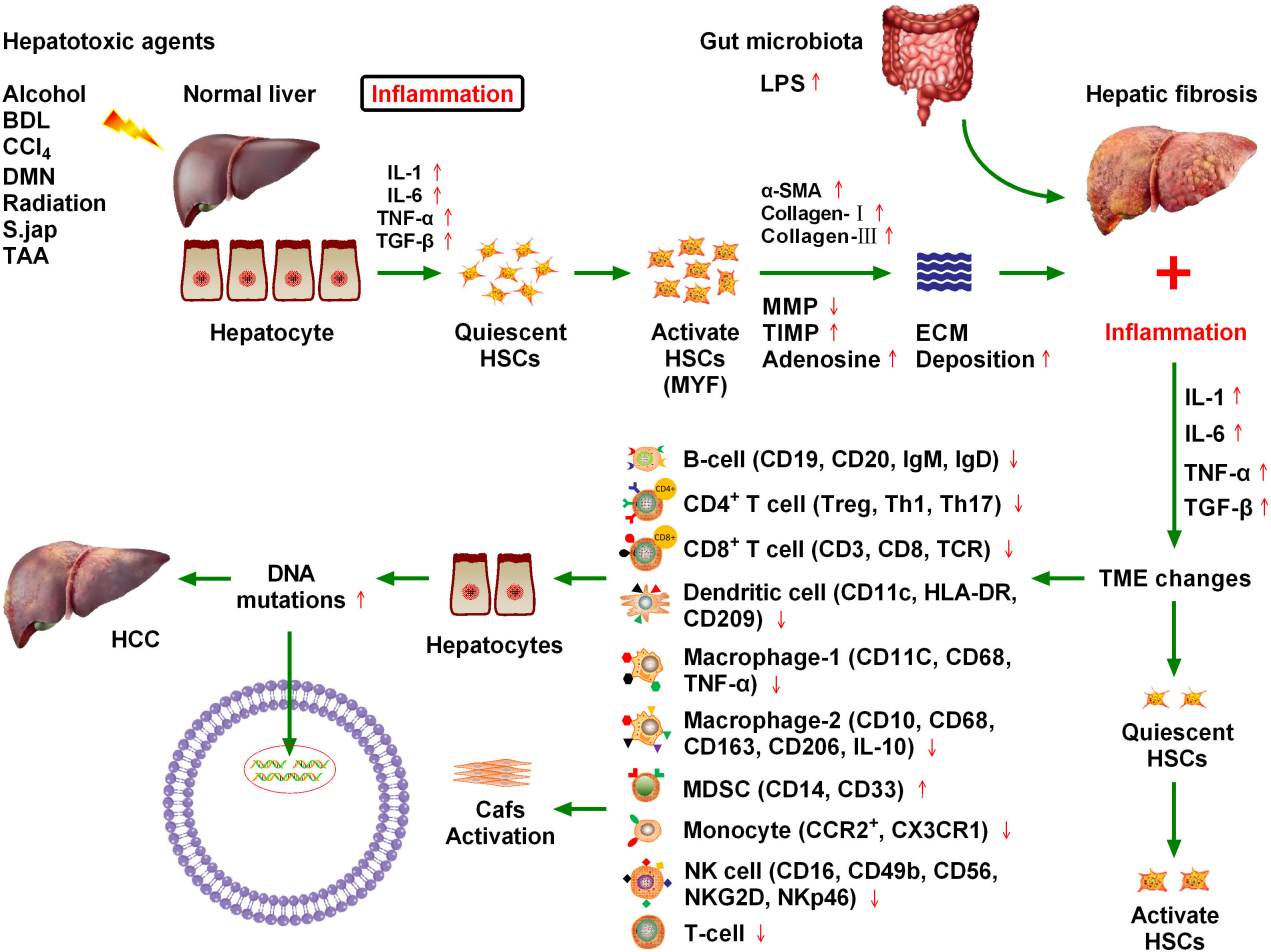
Chronic cytokine stimulation of liver cells leads to genetic mutations that induce hepatocellular carcinoma (HCC), and ultimately hepatic fibrosis. Various injury factors, including viral infection, alcohol, obesity, and insulin resistance, act on the liver, causing liver inflammation, an immune response, hypoxia, oxidative stress, hepatocyte necrosis, and apoptosis. Injury and inflammation cause regeneration of hepatocytes and activation of hepatic stellate cells (HSCs), which are the main sources of liver scarring. After activation, a series of phenotypic and biological behavior changes occur in HSCs, including the promotion of fibroplasia and migration and the release of many cytokines, which promote proliferation and angiogenesis, and prevent apoptosis. Inflammatory cells, such as macrophages, natural killer cell lymphoma and leukemia, T lymphocytes, and B lymphocytes, may be involved in liver injury and fibrosis. Chronic inflammation and regeneration processes lead to dysregulation of hepatocyte growth, genomic instability, DNA damage, dysplasia, and malignant transformation, which ultimately lead to HCC. The immune microenvironment composed of different immune cells plays a key role in HCC progression.

HCC is the fifth most common cancer worldwide and the third leading cause of cancer death (1). Recent epidemiological data indicate that death rates from HCC are increasing in the United States and Europe, and, together with incidence, are expected to double in the next 10–20 years (2). HCC can be primary or secondary. Primary HCC includes HCC and intrahepatic cholangiocarcinoma (ICC), mixed HCC, and bile duct cell carcinoma. HCC and ICC are the two main types of primary HCC (3). HCC is the most common primary liver cancer and the fourth leading cause of cancer-related death globally, accounting for more than 90% of all primary HCC cases. Chronic hepatitis B or C virus infection is the main cause of HCC, while other causes include alcoholism, autoimmune liver disease, and nonalcoholic steatohepatitis (4). Constant inflammation damages deoxyribonucleic acid (DNA) in regenerating liver cells, which leads to genetic changes that increase the chance of cancer development (5) (**Figure 1**). Chronic hepatitis infection or long-term liver injury often leaves the liver in a state of chronic inflammation (6, 7). Moderate inflammation can fight pathogens and repair tissue damage in liver, however, persistent liver inflammation can disrupt the microenvironment and tip the balance in favor of liver carcinogenesis (8, 9). Over the past decade, new researches have demonstrated that the immune microenvironment plays critical roles in HCC progression, and therapies targeting tumor microenvironment (TME) have been reported to effectively inhibit HCC growth in both animal models and clinical trials (6–9).

### 1.2 Hepatic fibrosis

Numerous inflammatory factors continue to stimulate hepatic stellate cells, leading to their activation, which is the main cause of hepatic fibrosis (10). Approximately 80% of HCC cases occur in the context of chronic inflammation and cirrhosis caused by viral hepatitis. The hepatic inflammation and fibrosis environment play an important role in the development of HCC, and the treatment and prognosis of HCC are also complicated by the tumor stage and the degree of liver dysfunction (11). Various injury factors, including viral infection, alcohol, obesity, and insulin resistance, act on the liver, which can cause liver inflammation, immune response, hypoxia, oxidative stress, hepatocyte necrosis, and apoptosis (12). Injury and inflammation cause the regeneration of hepatocytes and the activation of HSCs, which are the main sources of liver scars. After activation, HSCs undergo a series of phenotypic and biological behavior changes, including fibroplasia promotion, migration, and release of many cytokines that promote proliferation, angiogenesis, and anti-apoptosis (13). Inflammatory cells such as macrophages, natural killer (NK) cells, T lymphocytes, and B lymphocytes may be involved in liver injury and fibrosis. Chronic inflammation and regeneration processes lead to the loss of hepatocyte growth, regulatory genomic instability, DNA damage, dysplasia, and malignant transformation, ultimately leading to the development of HCC (13) (**Figure 1**).

### 1.3 Liver inflammation and the fibrosis microenvironment promote HCC progression

A close relationship exists between chronic liver inflammation, hepatic fibrosis, and HCC. Most chronic liver diseases are characterized by diffuse chronic inflammation, necrosis, and fibrosis. Chronic inflammation and fibrosis is a dynamic process of accumulation of lymphocytes, macrophages, and matrix cells that undergo secretory and paracrine interactions (11). Inflammatory cells belonging to innate immunity (e.g., NK cells and macrophages) and adaptive immunity (e.g., T lymphocytes and B lymphocytes) are involved in hepatic injury and fibrosis. By contrast, injured hepatocytes, Kupffer cells, and HSCs are involved in inflammation induction. Matrix cells can regulate the differentiation and function of antigen-presenting cells. The pattern of cytokine and chemokine secretion in the matrix determines T lymphocyte migration and polarization (14). Tumors may occur when chronic inflammation and injury healing processes become dysregulated. Malignant transformed hepatocytes may replace proliferative nodules, which are atypically altered during regeneration. In addition, hypoxia and inflammation are major factors that stimulate the proliferation of blood vessels and promote tumor growth (**Figure 1**). Inflammatory signals such as toll-like receptor 4 (TLR4) and nuclear factor kappa B (NFκB) promote tumor cell proliferation and migration by producing numerous cytokines and altering matrix, chemotactic growth, and lymphovascular hyperplasia factors (15). Because of the close relationship between hepatic fibrosis and HCC, it is crucial to explore the influence of fibrosis signaling on the occurrence, development, recurrence, and metastasis of HCC to improve the standard of HCC prevention and treatment (16).

## 2 Fibrotic signaling in HCC

**Figure 2:**
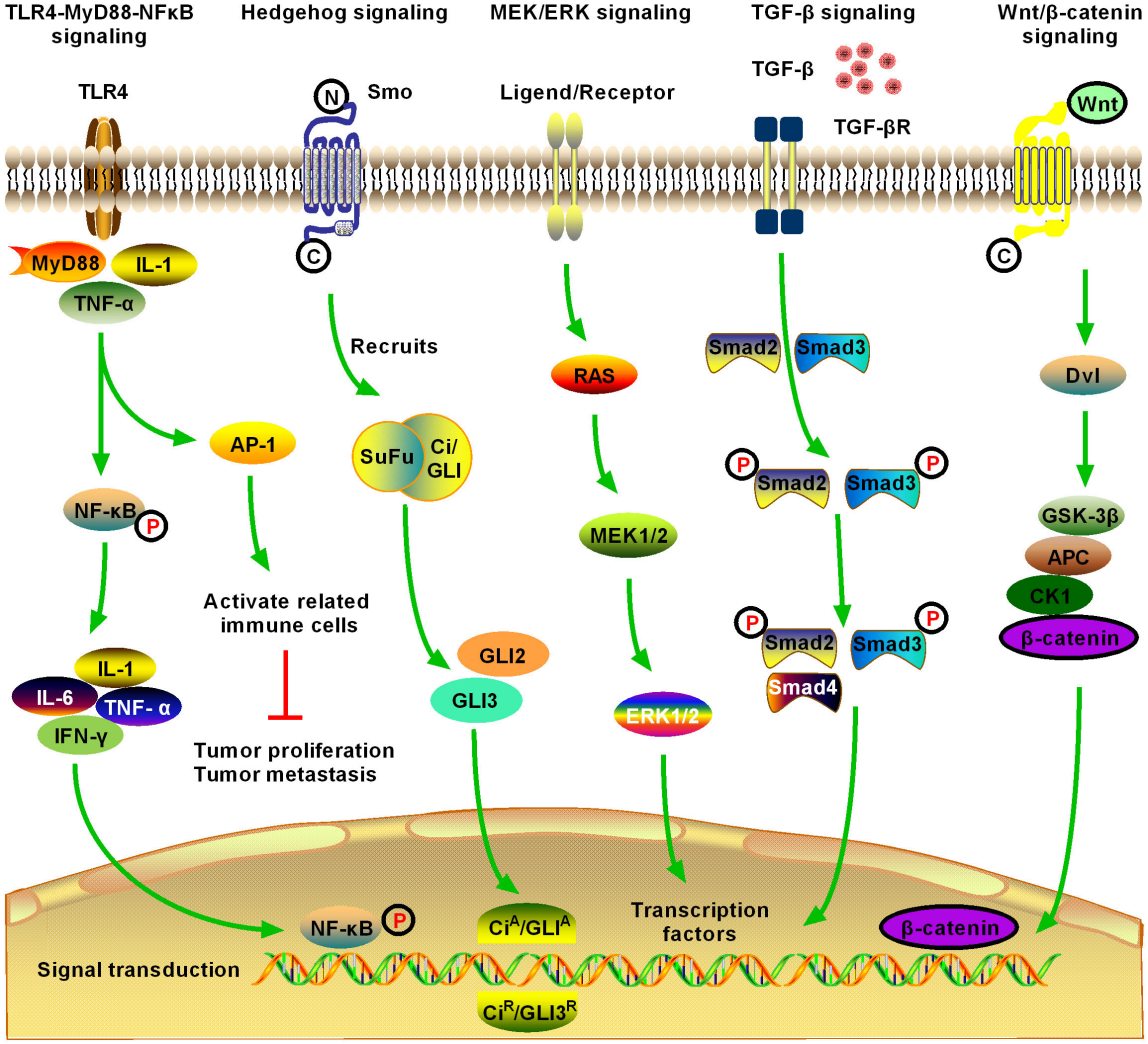
Schematic of five significant fibrotic signaling pathways in HCC, including toll-like receptor 4 (TLR4)-myeloid differentiation primary response gene 88 (MyD88)-nuclear factor kappa B (NFκB) signaling, Hedgehog (Hg) signaling, MAPK/extracellular signal-regulated kinase (MEK)/extracellular regulated protein kinase (ERK) signaling, transforming growth factor-β (TGF-β) signaling, and Wnt/β-catenin signaling (1). TLR4-MyD88-NFκB signaling is one of the most important inflammatory and fibrotic pathways discovered in recent years, the activation of which releases downstream inflammatory cytokines, inducing the production of interleukin (IL)-1, IL-6, and tumor necrosis factor (TNF)-α. Activation of NFκB downstream of the pathway is induced, and the signal enters the nucleus to induce activation, proliferation, invasion, and migration of HCC cells, and inhibit their apoptosis (2). Hg signaling is activated in different tumors and may contribute to the development of multiple tumor types by promoting the process of tumor initiation and metastasis. Novel Hg signaling inhibitors have entered the clinical research stage for the treatment of HCC; however, the development of Hg signaling targeted inhibitors still has broad prospects (3). MEK/ERK signaling is the most active research area in cell signal transduction recently. MEK/ERK signaling transfers a variety of extracellular signals to the nucleus through phosphorylation and activates various transcription factors, regulating cell proliferation, growth inhibition, differentiation, and apoptosis. MEK/ERK signaling is an important fibrosis signal in HCC (4). TGF-β1 is an important cytokine in the development of HCC, and it is also the strongest known fibrogenic factor. TGF-β1 signaling regulates the growth and proliferation of HCC cells. Currently, TGF-β1 is highly expressed in patients with HCC, where it is significantly correlated with the degree of tumor differentiation. The expression level of TGF-β1 increased with the decrease in tumor cell differentiation, suggesting that TGF-β1 can be used as an indicator for the early diagnosis of HCC. TGF-β signaling is an important fibrosis signaling of HCC (5). After activation of Wnt/β-catenin signaling, β-catenin accumulates continuously in the cytoplasm, which promotes part of β-catenin to enter the nucleus, activate and bind to the T cell factor/lymphoid enhancer transcription factor family, initiate the transcription of multiple downstream target genes, and promote the development of HCC. Activation of Wnt/β-catenin signaling promotes the activation and proliferation of HSCs and HCC cells and is an important fibrotic signaling pathway in HCC.

**Figure 3:**
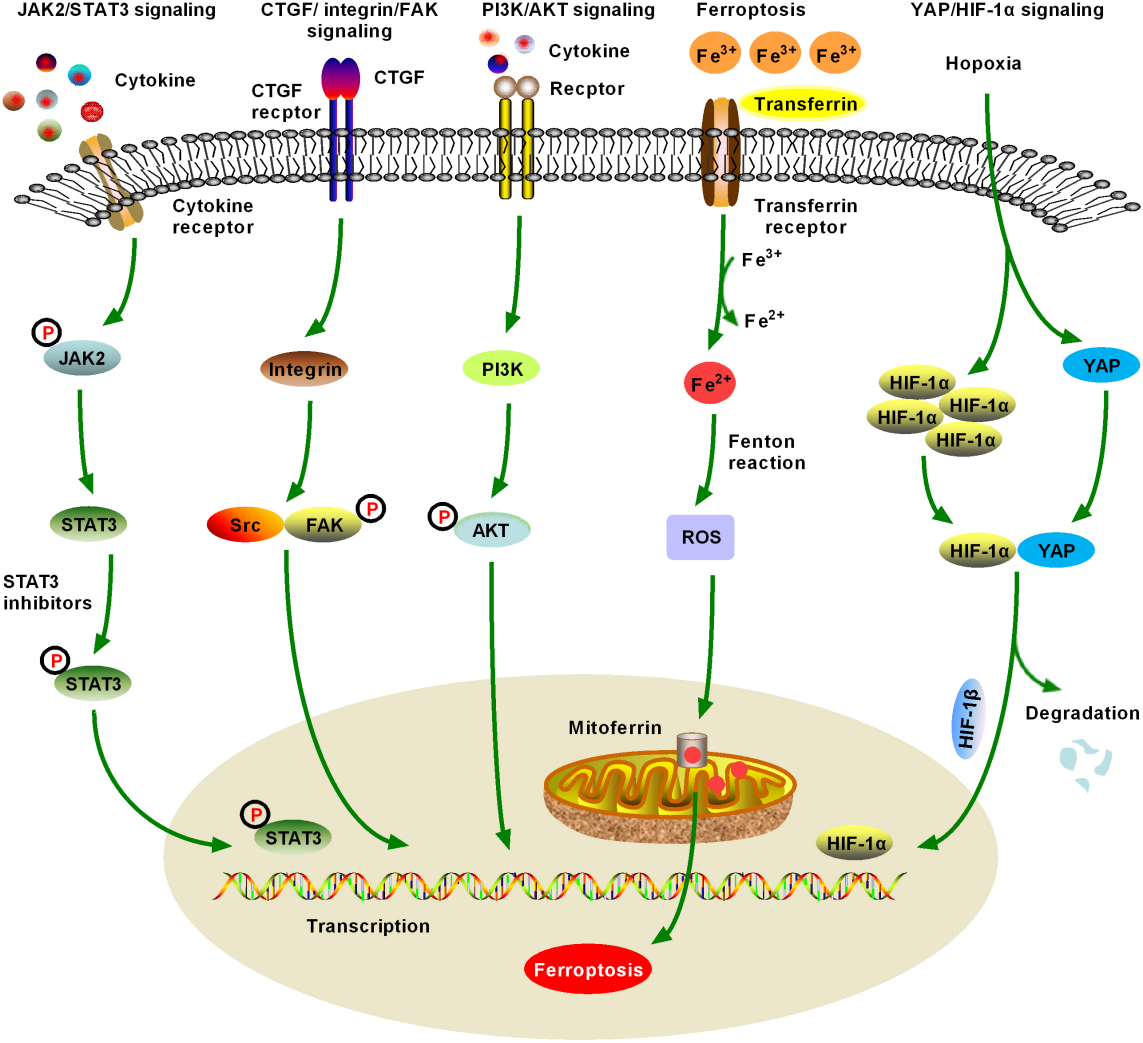
Schematic figure of another five significant fibrotic signaling pathways in HCC, including Janus kinase2 (JAK2)/signal transducer and activator of transcription 3 (STAT3), connective tissue growth factor (CTGF)/integrin/focal adhesion kinase (FAK), and Yes-associated protein (YAP)/hypoxia-inducible factor-1α (HIF-1α) signaling (6). JAK2/STAT3 signaling can be activated by various cytokines and growth factors, resulting in dysregulation of downstream target genes, which promote the formation and metastasis of malignant tumors by controlling cell proliferation, angiogenesis, immune surveillance, tumor invasion, and metastasis. JAK/STAT3 signaling is one of the important fibrotic signaling pathways in HCC (7). Increased expression and distribution of integrins have been observed in almost all human cancers, and multiple integrin-related genes are upregulated in HCC. Currently, crosstalk between CTGF/integrin/FAK and other signaling factors, such as TGF-β, has been extensively studied (8). Numerous studies have confirmed that the activation of phosphatidylinositol 3-kinase (PI3K)/AKT signaling promotes the proliferation, migration, and glycolysis of HCC cells, and is a widely studied fibrosis signal in the context of HCC. An increasing number of PI3K/AKT signaling-targeted inhibitors have been identified for preclinical or clinical trials (9). Ferroptosis was first proposed in 2012 and has quickly become a hot research topic. Various natural products improve hepatic fibrosis by inducing ferroptosis of HSCs and myofibroblasts and prevent HCC by inducing ferroptosis in HCC cells. However, as these studies are still in the initial stage and many research results are controversial, further studies are necessary (10). Hypoxia-induced YAP/HIF-1α signaling activation facilitates tumor cell growth, survival, and metastasis, and hypoxia is a central marker of HCC and its microenvironment. YAP/HIF-1α signaling is a widely studied fibrotic signaling in HCC. HIF-1α inhibitors may be developed as HCC therapeutic drugs in the future.

The HCC TME comprises matrix cells, including Kupffer cells, HSCs, cancer-associated fibroblasts (CAFs), liver sinusoids endothelial cells (LSECs), tumor-associated macrophages (TAMs), and lymphocytes. The HCC–HSC dialog plays an important role in the development of HCC (17). HSCs play a central role in the occurrence and development of ICC, especially in the cytokine dialog between ICC and matrix cells. Metastatic cancer cells enter the sinusoidal spaces of the liver, where most are captured and killed by Kupffer and NK cells (18). Escaped cancer cells form micrometastases and induce a microenvironment conducive to metastasis. These cells are activated following liver damage, after which, they differentiate into myofibroblast (MYF)-like cells and produce a large amount of cytokines, chemokines, growth factors, and extracellular matrix (ECM). In addition to producing and secreting collagen and other scar tissue, HSCs have other important functions, including participating in liver regeneration, immune regulation, immune tolerance, and liver tumorigenesis. Various cytokines mediate the development of HCC through multiple fibrosis signaling pathways (19). Therefore, here we review recent advances in the field of hepatic fibrosis and HCC and consider several molecular signaling pathways that contribute to HCC in the hepatic fibrosis microenvironment (20) (**Figures 2**, **3**, **Table 1**).

**Table 1:** Major drugs targeting the 10 signaling pathways.

Targeting signaling	Agonist/Antagonist	Drugs	Characteristics	References
TLR4-MyD88-NFκB Hedgehog MEK/ERK TGF-β Wnt/β-catenin JAK2/STAT3 CTGF/integrin/FAK PI3K/AKT Ferroptosis YAP/HIF-1α	(1) Antagonist (1) Hg antagonist (2) Smo antagonist (3) Gli antagonist (1) Antagonist Antagonist Inhibitor Inhibitor Integrin Antagonist (1) Inhibitor (2) Agonist (1) Inducer (2) Inhibitor (1) Inhibitor	① Polysaccharide of Atractylodes ② Ginkgolide-A ① IPI-926 ② Vismodegib ① Erismodegib (NVPLDE225) ② BMS-833923 (XL139) ③ IPI-926 ① As_2_O_3_ ② Imiquimod ③ RU-SKI 43 ④ GANT-61 ⑤ GANT-58 ⑥ FN1-8 ① NVP-AAL881 ② Sorafenib ③ PD098059 ④ UO126 ⑤ Tanshinol ① Physalin D ② Oxymatrine ③ Galunisertib ④ Fresolimumab ⑤ 1D11 ⑥ EMD527040 ⑦ Cilengitide ⑧ NIS793 ① ICG-001 ② PRI-724 ③ OMP-54F28 (Ipafricept) ④ XAV939 ⑤ OMP-54F28 ⑥ sFZD7 ⑦ DKN-01 ⑧ PKF115-548 ⑨ PKF222-815 ⑩ MG132 ① AG490 ② NSC74859 ③ Sorafenib ④ SP600125 ⑤ Danshensu ⑥ Genistein ⑦ Mg isoglycyrrhizinate ⑧ Mangiferin ⑨ Cantharidin ⑩ Etomidate ⑪ AZD1480 ⑫ Cucurbitacin I ⑬ FLLL32 ⑭ S3I-201 ⑮ Toosendanin ⑯ Y705 ⑰ BP-1-102 ⑱ AZD1480 ① CWHM-12 ② abciximab ③ Eptifibatide ④ Tirofiban ⑤ EMD 525797 ⑥ CNTO-95 ⑦ EMD121974 ⑧ GLPG0187 ⑨ CWHM12 ⑩ MK-0429 ⑪ IDL-2965 ⑫ STX-100 ⑬ GSK3008348 ⑭ PLN-1474 ⑮ PLN-74809 ⑯ BG00011 ⑰ Natalizumab ⑱ IDL-2965 ⑲ PLN-1474 ⑳ Abciximab 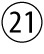 Natalizumab 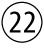 Vedolizumab 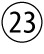 Lifitegrast ① Curcumin ② MK2206 ③ LY294002 ④ Kinsenoside ⑤ Maltitol ⑥ Cytisine derivatives ⑦ Salvianolic acid A ⑧ Aloperine ⑨ BYL-719 ⑩ GDC-0941 ⑪ Apatinib ⑫ Astragalin ⑬ Jolkinolide B ⑭ Oxaliplatin ⑮ Bicyclol ⑯ Arenobufagin ⑰ Peurarin ⑱ Aloin ⑲ BEZ235 ⑳ Betulinic acid 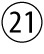 Isoviolanthin 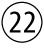 Ginsenoside Rk3 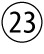 GSK690693 ① Doxazosin ② Quercetin ① Erastin ② Sorafenib ③ RSL3 ④ Sulfasalazine ⑤ RSL3 ⑥ RSL5 ⑦ DPI compounds ⑧ FIN56 ⑨ CIL56 ⑩ Statins ⑪ FINO2 ⑫ 1,2-dioxolane ⑬ Artesunate ⑭ Berberine ⑮ Ellagic Acid ⑯ Mg isoglycyrrhizinate ⑰ Chrysophanol ⑱ Wild bitter gourd extract ⑲ Altretamine ⑳ ML-162 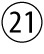 Dihydroartemisinin 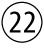 Artemether 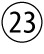 Sulfoximine Buthionine 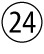 #xa0;Lanperisone 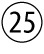 #xa0;Acetaminophen 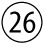 #xa0;Cisplatin 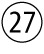 #xa0;Aferin A ① Deferoxamine Mesylate ② Liproxstatin-1 ③ Ferrostatin-1 ④ Vitamin E ⑤ 2-mercaptoethanol ⑥ Cycloheximide ⑦ Rosiglitazone ⑧ Pioglitazone ① Curcumin ② PX-478 ③ Metformin ④ Nicotinamide mononucleotide ⑤ Oroxylin A ⑥ LW6 ⑦ Bruceine D ⑧ 32-134D	① Most are in clinical studies ② Most are natural products ① Treatment of some cancers ① Lack specificity ② Susceptible to drug resistance ① Inhibited Hg target gene Sufu expression ② Good drug resistance ① Effective against HCC ② Effective against advanced liver cancer ① Numerous research works at present ② Research hotspot ③ Many research institutions are researching ① Most are in clinical studies ② Broad prospects ③ Many research institutions are researching ④ More varieties ⑤ Lack specificity ① Research hotspot ② Most are in clinical studies ③ Broad prospects ④ Includes many natural products ⑤ Numerous varieties ① Excellent potential ② Poor targeting ③ Many products have high toxicity ④ Many varieties used in research ⑤ Broad prospects ⑥ Includes many natural products ⑦ Most in thepreclinical research stage ① Currentresearch hotspot ② Poor targeting ③ Combination medication required ④ Includes many natural products ⑤ Drugs alreadyon the market ⑥ Many clinical studies underway ⑦ Various types ① Controversial ② Needs further research ① Recently discovered and reported ② Currentresearch hotspot ③ Requires further research ④ Numerous types ⑤ Includes many natural products ⑥ Controversial ⑦ Mechanism research is imperfect ① Controversial ② Mechanism research is imperfect ③ Requires further research ④ Discovered late ① Currentresearch hotspot ② Discovered late ③ Requires further research ④ Mediates various liver diseases ⑤ Little variety	(21) (22) (23) (24) (25, 26) (27) (28) (29) (30, 31) (32) (33) (34) (35, 36) (37) (38) (39, 40) (41, 42) (43, 44) (45, 46) (47) (48) (49) (50) (51) (52) (53) (54, 55) (56, 57) (58, 59) (60, 61) (62, 63) (64, 65) (66, 67) (68) (69) (70) (71) (72) (73, 74) (75) (76) (68) (67) (72) (77, 78) (79, 80) (81, 82) (83, 84) (85, 86)

### 2.1 TLR4-MyD88-NFκB signaling pathways

Activation of the TLR4-myeloid differentiation primary response gene 88 (MyD88)-NFκB signaling pathway, an important pathway associated with the inflammatory response and hepatitis/hepatitis fibrosis, can lead to the release of downstream inflammatory factors and induce the production of interleukin (IL)-1, IL-6, and tumor necrosis factor (TNF)-α. TLR4 has been shown to pass through the Toll/IL-1 receptor domain (TIR domain) and use the TIR domain adaptor proteins MyD88, IL-1 receptor-associated kinase, and TNF receptor-s-sociated factor 6, to induce downstream NFκB activation to produce inflammatory factors (87). Extracellular inflammatory signals are presented to MyD88 via the TLR4-mediated signaling pathway, which activates NFκB, inducing its nuclear translocation and subsequent signal transduction (88) (**Figure 2**).

#### 2.1.1 TLR4-MyD88-NFκB signaling and hepatic fibrosis

Hepatic fibrosis is the result of long-term chronic liver injury and repeated scar repair, which is mainly characterized by excessive deposition of ECM. HSCs are the main source of scar tissue in hepatic fibrosis. HSCs possess a complete TLR4 signaling pathway, which mediates important biological characteristics, including inflammation phenotype tolerance to apoptosis in fibrosis. Indeed, the degree of fibrosis in mice with *TLR4* mutation has been shown to be significantly reduced in the three hepatic fibrosis models of bile duct ligation (BDL), carbon tetrachloride (CCl_4_), and thioacetamide (TAA), indicating that the TLR4 signaling pathway is involved in the occurrence of hepatic fibrosis (89, 90). Moreover, lipopolysaccharide (LPS) has been shown to cause swelling of hepatocytes, disappearance of the hepatic cord structure, and infiltration of numerous inflammatory cells. LPS has also been shown to upregulate the messenger ribonucleic acid (mRNA) and protein expression levels of TLR4, MyD88, IκBα, and NFκB, and increase the secretion of cytokines, including IL-1β, IL-4, IL-6, and TNF-α levels. Guo et al. (21) found that pretreatment with polysaccharide of Atractylodes macrocephala Koidz (PAMK) relieved LPS-induced histopathological damage in mice, and could activate TLR4-MyD88-NFκB signaling, reduce the levels of IL-1β, IL-6, and TNF-α, increase IL-4 levels, and inhibit the levels of glutathione peroxidase and malondialdehyde. These results indicate that PAMK could reduce inflammatory damage and oxidative stress in mice and play a protective role in the early stages of LPS invasion of the liver. However, overexpression of the TLR4/MyD88/NFκB axis and its downstream pro-inflammatory mediators, such as TNF-α, IL-6, and interferon (IFN)-γ, were observed in mice with CCl_4_-induced cirrhosis. Inhibiting the TLR4/MyD88/NFκB signaling pathway has protective effects on liver injury induced by various inflammatory cytokines (22). Activation of the TLR4-myD88-NFκB signaling pathway, an important pathway associated with hepatic fibrosis and HCC, can lead to the release of downstream inflammatory factors and induce the production of IL-1, IL-6, and TNF-α. These results suggest that inhibition of the TLR4-MyD88-NFκB-mediated inflammatory response can down-regulate the expression of TLR4 mRNA and protein, thereby improving hepatic fibrosis (91) (**Table 1**).

#### 2.1.2 TLR4-MyD88-NFκB signaling pathway and HCC

At present, there are at least two views on the mechanism by which the TLR4-mediated signaling pathway induces HCC formation. The TLR4-MyD88-dependent signaling pathway can activate transcription factors such as *NFκB* and *activatorprotein-1* (*AP-1*), before activating related immune cells to play the role of immunosuppression and promote the development of tumor disease (92). It has been reported that intraperitoneal injection of diethylnitrosamine (DEN) to induce HCC in animals activates the TLR4-mediated MyD88 signaling pathway, resulting in the activation of Kupffer cells, the production of IL-6 and other pro-inflammatory mediators, and the induction of carcinogenic effects of the TLR4-MyD88-dependent pathway (93). Therefore, the activation of the TLR4-MyD88 signal is considered to be one of the important causes of HCC. In-depth studies on HCC have shown that the downstream multifunctional NFκB signaling pathway regulated by TLR4 plays a key role in the induction of tumor formation by inflammatory mediators. TLR4-MyD88-NFκB signaling plays a positive regulatory role in the inflammatory progression of HCC, suggesting that the TLR4-MyD88-NFκB signaling pathway may be a new target for the prevention or treatment of HCC (94). The regeneration of hepatocytes caused by inflammatory damage induced by the activation of TLR4-MyD88-NFκB signaling pathway mostly originates from the activation of HSCs, which lead to various biological changes, including promoting the proliferation and migration of fibrous tissue and secreting several cytokines with antiapoptotic effects and the ability to promote proliferation. Chronic inflammation leads to abnormal cell growth, gene expression disorders, and DNA damage in liver tissues, which further results in malignant tissue development and ultimately induces HCC.

### 2.2 Hedgehog signaling pathway

The discovery of the Hedgehog (Hg) signaling pathway stems from studies of embryonic development in Drosophila melanogaster. In humans, the pathway transmission process can be simply summarized as the Hg Homo sapiens *patched 1-Smoothened-Glioma associated oncogene homolog* (*Ptch1*–*Smo*–*Gli*) process. As the most important nuclear transcription factor in the Hg pathway, *Gli-l* is responsible for regulating many downstream effector factors of the pathway and binds to the promoter of downstream genes in the Hg signaling pathway to directly regulate the transcription and expression of target genes (**Figure 2**). Recent studies have shown that the Hg pathway is abnormally activated in various liver diseases (95, 96). In addition, the Hg signaling pathway can promote the proliferation of tumor cells, inhibit apoptosis, and promote the occurrence of HCC. Therefore, the Hg signaling pathway is closely related to hepatic fibrosis and HCC (97). The Hg signaling pathway not only participates in cell growth and differentiation but also plays an important role in tissue and organ damage repair and immune regulation. However, abnormal activation of the Hg signaling pathway can also lead to abnormal development and even tumors.

#### 2.2.1 Hg signaling and hepatic fibrosis

Many studies have shown that the immune cell-mediated microenvironment of hepatic fibrosis is closely related to the activation of the Hg signaling pathway (98). Natural killer T (NKT) cells also produce Sonic Hedgehog (SHH), promote collagen secretion, and transform stationary HSCs into MYFs, resulting in hepatic fibrosis (99, 100). The human Hg protein is highly expressed in stationary HSCs, and the key gene of the Hg signaling pathway (*Gli-1 Gli-2*) cannot be detected. However, after 24 h of culture, the expression of human Hg interacting protein in HSCs decreased by 90% and the Hg signaling pathway ligands SHH and Gli-1 increased significantly, leading to activation of the Hg signaling pathway (101). The pathological characteristics of hepatic fibrosis include excessive synthesis and insufficient degradation of ECM, leading to its deposition in the liver. Persistent liver fibrosis may develop into cirrhosis and increase the risk of HCC. Inhibition of the Hg pathway (using Smo antagonists or by knocking out the *Smo*) can reduce the activation of quiescent HSCs, reduce the production of MYF HSCs, and reduce the degree of hepatic fibrosis (**Table 1**). Although the Hg signaling pathway is associated with quiescent HSC activation, the activation mechanism is unclear. Although the Nobel Prize winners Wieschaus and Nussland-Volhart reported the Hg pathway in 1980, its importance in dictating hepatic fibrosis and HCC outcomes has emerged much more recently (102). Fan et al. found that suppressing the activation of HSCs could alleviate hepatic fibrosis in mice induced by CCl_4_ and a 3,5-diethoxycarbonyl-1,4-dihydrocollidine diet for 8 weeks via the Hg pathway (103). In mice or patients with hepatic fibrosis, inhibition of glutaminase blocked the accumulation of MYFs and fibrosis progression. Du et al. reported that knockout of the Hg signaling intermediate heptahelical transmembrane G protein-coupled receptor SMO or knockdown of Yes-associated protein (YAP) inhibited the expression of glutaminase, the rate-limiting enzyme in glutaminolysis, in mice and patients with hepatic fibrosis (99). Hg signaling regulates glutaminolysis to inhibit HSC activation. *In vivo* and *in vitro* studies confirmed that procyanidin B2, a flavonoid extract that is abundant in grape seeds and has pharmacological effects, alleviated CCl_4_-induced hepatic fibrosis in mice by inhibiting the activation of HSCs and angiogenesis via the Hg pathway during hepatic fibrosis (98). In other words, the Hg pathway plays an important role during various liver injuries, such as hepatic fibrosis, inflammation-related injury, and liver carcinogenesis. Targeting the Hg pathway has become a promising treatment for hepatic fibrosis.

#### 2.2.2 Hg signaling pathway and HCC

Abnormal activation of the Hg signaling pathway is closely related to the invasion and metastasis of malignant tumors. Aberrant activation of Hg signaling in the normal liver and HCC has been demonstrated in detail in previous studies (23). A new study found that the stage of hepatic fibrosis was associated with the degree of Hg signaling activation in patients with non-alcoholic fatty liver disease (NAFLD). Activation of Hg signaling is also associated with fibrosis in the lungs, skin, and kidneys (104). Chung et al. further investigated up-regulated hepatic expression of SHH-induced hepatic fibrosis and hepatocarcinogenesis in a transgenic mouse model (105). GANT61 (NSC136476) is a *Gli-1*- and *Gli-2*-induced transcriptional inhibitor that inhibits Hg. Wang et al. found that GANT61 significantly suppressed Hg signaling to reverse sorafenib resistance in CD44-positive HCC tissues (97). This combined administration may be effective in patients with HCC with high CD44 levels as a personalized medicine approach. Another study has shown that the Hg pathway in liver tissue of Chinese patients with HCC is activated by ligand expression, rather than by mutations, suggesting that the research prospects of Hg signaling inhibitors are very broad for a large number of patients with HCC in China (24). Currently, the role of the Hg signaling pathway in liver physiology and pathology has not been fully elucidated and requires further study.

### 2.3 MEK/ERK signaling pathway

HSC activation is facilitated by several mitogen-activated protein kinases (MAPKs), including MAPK/extracellular signal-regulated kinase (MEK), extracellular regulated protein kinase (ERK), connective tissue growth factor (CTGF), and insulin-like growth factor-1 (106). MEK binding to ERK results in dimerization of tyrosine (Tyr) residues, which activates the phosphatidylinositol 3-kinase (PI3K) and MAPK pathways. Blocking MEK/ERK signaling blocks MAPK and PI3K/AKT signaling pathways, thereby inhibiting HSC activation and attenuating experimental hepatic fibrosis progression (**Table 1**). ERK/MAPK signaling pathways are activated and involved in cell growth, differentiation, and migration during the pathogenesis of hepatic fibrosis, cirrhosis, and HCC (25, 107). The MEK/ERK signaling pathway is closely related to hepatic fibrosis and HCC (**Figure 2**).

#### 2.3.1 MEK/ERK signaling and hepatic fibrosis

Studies have confirmed that ERK is a major member of the MAPK family and that its MEK/ERK signaling pathway is an important pathway for many cytokines to regulate cell proliferation and apoptosis. Foglia et al. suggested that inhibiting the MEK/ERK pathway in activated MYF-like HSCs is a key crossroad for reversing hepatic fibrosis (26). HSC activation by myofibroblastic differentiation is critical for hepatic fibrosis. Crosstalk between HSCs/myofibroblastic and tumor cells in the microenvironment alters the properties and facilitates the growth, proliferation, migration, and invasion of HCC cells. Homo sapiens *E2F transcription factor 3* (*E2F3*) acts as a transcriptional activator and increases cell proliferation through G1/S transformation. A new report has identified a novel stiffness-mediated HSC activation mechanism that is dependent on the *E2F3* (108). Liu et al. found that HCC cells cultured in an HSC-conditioned medium activated the PI3K/AKT and MEK/ERK signaling pathways following the combination of *E2F3* with the *fibroblast growth factor 2* (*FGF2*) promoter, which increased the growth and metastasis of HCC cells. In addition, gene knockout of *E2F3* mitigated DEN- and CCl_4_-induced HCC in mice. The *E2F3* is also highly expressed in the HCC tissues of patients; therefore, matrix stiffness modulates HSC activation into tumor-promoting MYFs via *E2F3*-dependent MEK/ERK signaling and regulates malignant progression. The ERK pathway is critical for transducing signals from surface receptors to the nucleus and is overactivated in many tumors, including HCC, melanoma, and breast cancer.

#### 2.3.2 MEK/ERK signaling and HCC

According to new statistics, the majority (approximately 80%) of HCC cases result from severe hepatic fibrosis and/or cirrhosis. Li et al. found that the serum cartilage oligomeric matrix protein (COMP) levels in patients with HCC were obviously higher than those in healthy people, and these patients showed more unfavorable disease parameters, including a higher incidence of vascular invasion and HCC recurrence (106). In addition, gene knockout animal experiments and different cell experiments demonstrated for the first time that COMP mainly originates from activated HSCs and promotes the growth and metastasis of HCC cells in a dose-dependent manner by activating MEK/ERK and PI3K/AKT signaling. Moreover, crosstalk was observed between hepatic fibrosis and HCC through inhibiting ERK signaling, which is a potential novel target for the prevention and treatment of HCC. Another study showed that low-density lipoprotein receptor inhibited the enhancement of intracellular cholesterol synthesis through MEK/ERK signaling and promoted the proliferation and metastasis of HCC cells (109). It has also been reported that Homo sapiens minichromosome maintenance complex component 6 promotes HCC metastasis through MEK/ERK signaling and can be used as a novel serum biomarker for early recurrence (110). Lai et al. found that NEI-like DNA glycosylase III (NEIL3) activation of MEK/ERK signaling mediated epithelial–mesenchymal transition (EMT), that treatment resistance promoted HCC progression, and that NEIL3 induction of targeted inhibition of NEIL3 was a promising therapeutic approach in patients with HCC (111). Currently, sophoridine derived from natural products has been found to inhibit the growth of lenvatinib-resistant HCC by inhibiting rat sarcoma virus (RAS)/MEK/ERK signaling by decreasing vascular endothelial growth factor receptor 2 expression (25). Another natural product, Morusinol, inhibits HCC cell invasion and migration and targets RAS/MEK/ERK signaling by inducing autophagy, and has selective and effective antitumor activity against human HCC (112). These studies further confirm that inhibition of MEK/ERK signaling alleviates HCC.

### 2.4 TGF-β signaling pathway

Discovered by Tucker in 1984, transforming growth factor-β (TGF-β) is a polypeptide cell growth regulatory factor associated with the growth of various tumors. The TGF-β superfamily members include at least five isomers (TGF-β1, 2, 3, 4, 5), among which TGF-β1 is the most closely related to liver injury and disease occurrence (113). TGF-β1 is a secretory polypeptide factor, which has various biological activities, including regulating cell growth, migration, differentiation, the occurrence and development of embryos and tumors, wound healing, bone formation, and immune regulation. Normal hepatocytes either lack TGF-β1 or show low levels (114). Meanwhile, damaged liver endothelial cells cause platelets to agglutinate and release TGF-β1. Studies have shown that TGF-β1 is a crucial factor leading to hepatic fibrosis and even HCC in the process of liver injury. The drosophila mothers against decapentaplegic protein (Smad) is the substrate of the most important intracellular kinase of the TGF-β1 receptor known to date. Activated TGF-β1 receptors recruit Smads through Smad anchor proteins or directly bind to signal molecules such as MAPK and phosphorylate them, so that signals are transmitted step by step in the cell until they are transferred to the nucleus, where they regulate the expression of target genes (115) (**Figure 2**).

#### 2.4.1 TGF-β signaling and hepatic fibrosis

Most literature studies have shown that TGF-β1 stimulates HSC activation and proliferation, leading to hepatic fibrosis (27, 28, 116, 117) (**Table 1**). TGF-β1 in the liver is mainly secreted by immune cells, HSCs, and epithelial cells, mainly through mediating the activation of HSCs to produce excessive ECM, leading to hepatic fibrosis (117). Xiang et al. reported that Physalin D alleviated CCl_4_- and BDL-induced hepatic fibrosis in mice via blocking TGF-β/Smad signaling and reducing HSC activation, proliferation, and transformation (27). Compound kushen injection, an approved traditional Chinese medicine formula, reduced the inflammatory response, oxidative stress, liver compensatory proliferation, and hepatocellular death of mice with hepatic fibrosis induced by CCl_4_ injection or a methionine choline-deficient diet via rebalancing TGF-β/Smad7 signaling in HSCs, which protected against hepatic fibrosis and hepatocarcinogenesis in both preclinical and clinical studies (28). Sulfatase-2 (Sulf2) also regulates hepatic fibrosis in mice induced by BDL or intraperitoneal injection of CCl_4_ or TAA through inhibiting TGF-β signaling (118). *Sulf2* knockout (*Sulf2*-KO) mice showed significantly decreased collagen content and bands of bridging fibrosis compared with wild-type mice in all three models of hepatic fibrosis. Sulf2 expression has recently been shown to be upregulated in cirrhotic human liver and fibrotic mouse liver, suggesting that Sulf2 plays an important role in both fibrosis and subsequent tumorigenesis. TGF-β is highly expressed in tissues of hepatic fibrosis and HCC, so the TGF-β signaling is considered to be a marker of hepatic fibrosis and HCC. The synergistic role of TGF-β and the tissue microenvironment in modulating the cellular response of different cell types and promoting the development of hepatic fibrosis and the progression of HCC has been extensively demonstrated (29). TGF-β signaling provides a wide scope for intracellular crosstalk, such as receptor-associated Smads interacting with other signaling molecules rather than directly transmitting signals to the nucleus, and the activation of intracellular substrates other than Smad can be mediated by influencing apoptosis and other activation of other intracellular signaling pathways (119). The mechanism of treating hepatic fibrosis through targeted inhibition of TGF-β signaling has been widely discussed (119, 120).

#### 2.4.2 TGF-β signaling and HCC

TGF-β1 is an important cytokine in the occurrence and development of HCC. As well as being an important cytokine that controls the growth and proliferation of hepatocytes, TGF-β1 is also known to be highly expressed in patients with HCC and is significantly correlated with the degree of tumor differentiation (29). Recently, cancer-associated fibroblast-mediated cell crosstalk supporting HCC progression has become a research hotspot (121). CAFs are key players in the pathogenesis of HCC; however, the complex mechanism of crosstalk between CAFs and other components of the TME is still unclear, and studies on TGF-β1 activation of CAFs are ongoing (121). Aberrant activation of TGF-β/Smad signaling facilitates tumor metastasis and is often observed in HCC. The lncRNAlnc-UTGF has been shown to mediate a positive feedback loop regulates TGF-β/Smad signaling and promotes hepatoma metastasis (122). Moreover, bone morphogenetic protein (BMP) is involved in TGF-β signaling crosstalk; indeed, Ning et al. found that TGF-β1/BMP-7 pathway imbalance induced by activation of liver polarized macrophages promotes the aggressiveness of HCC (123). Increasing evidence has shown that TGF-β signaling plays a critical role in the regulation of immune cells, including CD4+ T cells, CD8+ T cells, dendritic cells (DCs), NK cells, myeloid suppressor cells, and TAMs. TGF-β signaling mediates HCC progression through the critical regulation of various immune cells in the liver to maintain a balance between immune tolerance and activation (124). *RALYL*, a liver progenitor-specific gene, was recently found to promote tumorigenicity, self-renewal, chemotherapy resistance, and metastasis of HCC by up-regulating TGF-β signaling and subsequent PI3K/AKT and signal transducer and activator of transcription 3 (STAT3) signaling to enhance HCC stemness (125). Increasing evidence suggests that crosstalk between ECM and TGF-β, and intracellular TGF-β signaling often exhibits crosstalk with other signals, including Jun N-terminal kinase, p38, MAPK, and NFκB, to regulate hepatic fibrosis and HCC (126). Some studies have explored the underlying molecular mechanisms of STAT3 crosstalk with Smad3/TGF-β1 signaling during EMT in patients with HCC and a rat model. Both *in vivo* (HCC patients, DEN-induced HCC rat model) and *in vitro* (HepG2, Bel7402, MHCC97H, and HCCLM3) results indicate that TGF-β1-induced EMT is dependent on Smad3-mediated Snail transcription and crosstalk of STAT3 signals in HCC cells (30). Interestingly, new studies have shown that TGF-β has a dual role in tumorigenesis, acting as a tumor suppressor in the early stages of tumorigenesis and promoting tumor progression and metastasis in more advanced cancers (31). In addition, the crosstalk between TGF-β and vascular endothelial-derived growth factor (VEGF) signals in a variety of immune cells, tumor cells, and matrix cells may further promote TGF-β-mediated immunosuppression, which may be a new mechanism for TGF-β to regulate tumor immune escape through immunosuppression in the latest tumor stage (126). However, TGF-β1 plays a role in promoting tumor growth and metastasis in the middle and late stages of the tumor. Overexpression of TGF-β1 in HCC cells does not inhibit the proliferation of HCC cells, which may be related to the defective receptor.

### 2.5 Wnt/β-catenin signaling

Wnt/β-catenin signaling comprises a group of evolutionarily conserved signals that play an important role in cell genesis, embryonic development, cell proliferation and differentiation, and tissue regeneration in various organisms (35). Recent studies have shown that Wnt signaling can participate in the activation of HSCs and the formation of hepatic fibrosis, which is mainly manifested in promoting collagen synthesis and collagen deposition in the liver by means of autocrine and/or paracrine mechanisms, thus reducing the apoptotic capacity of activated HSCs (36) (**Table 1**). Various stimulators can also play a role through the classical Wnt/β-catenin signaling pathway, ultimately promoting the occurrence and development of hepatic fibrosis. Other studies have found that abnormal activation of Wnt/β-catenin signaling is closely related to the progression and carcinogenicity of malignant tumors (127) (**Figure 2**). Specifically, the expression level of intracellular β-catenin is closely related to tumor metastasis and invasion of blood vessels and may lead to tumor enlargement. Wnt/β-catenin signaling is closely related to hepatic fibrosis and HCC.

#### 2.5.1 Wnt/β-catenin signaling and hepatic fibrosis

Activation of classical Wnt/β-catenin signaling can regulate cell proliferation, survival, and behavior. New studies have found that a series of intracellular signals drive HSC activation, including Wnt/β-catenin signaling, TGF-β/Smad signaling, and Hg signaling, with complex crosstalk between them (32). Increasing evidence suggests that inhibition of Wnt signaling to β-catenin may alleviate hepatic fibrosis; however, further research is needed to determine the identity and cellular origin of the factors that activate β-catenin in HSCs (32). Liu et al. found that insulin-like growth factor binding prote-3, Dickkopf-3 (DKK-3), and DKK-1 derived from human amniotic mesenchymal stem cells (MSCs) mitigate hepatic fibrosis via suppression HSC activation through blocking Wnt/β-catenin signaling in mice induced by injecting CCl_4_ via the tail vein. In addition, crosstalk between PI3K/AKT and Wnt/β-catenin signals were found during HSCs (LX-2) transfection assays in underlying mechanism studies (128). Rong et al. found that human bone marrow MSC-derived exosomes reduced CCl_4_-induced hepatic fibrosis in mice through Wnt/β-catenin signaling via both cellular assays and animal experiments (129). Upstream regulatory mechanisms that inhibit hepatic Wnt/β-catenin activity may constitute targets for the development of new therapies against life-threatening diseases such as hepatic fibrosis and HCC (130). Therefore, the regulation of β-catenin is expected to become a target of anti-hepatic fibrosis therapy, as well as a direction of future research.

#### 2.5.2 Wnt/β-catenin signaling and HCC

Numerous literature studies have shown that inhibition of Wnt/β-catenin signaling can inhibit HCC cell differentiation and proliferation and promote apoptosis during HCC progression (33, 131, 132). TAMs are an important component of the TME mediating the development of HCC. Yang et al. confirmed for the first time that the typical Wnt/β-catenin signaling crosstalk between HCC cells and macrophages promotes the polarization of M2-like macrophages, thus leading to tumor growth, migration, metastasis, and immunosuppression in HCC (133). Therefore, blocking Wnt/β-catenin signaling activation in HCC cells secreting Wnt and/or TAMs may be a potential strategy for future HCC treatment. Up to 70% of patients with HCC show up-regulation of Wnt/β-catenin signaling, and β-catenin mutations increase with HCC progression (134). Wang et al. reported that brain-expressed X-linked protein 1 (BEX1) plays a critical role in regulating cancer stem cell (CSC) properties in different types of HCC; indeed, targeting BEX1-mediated Wnt/β-catenin signaling may help to address the high recurrence rate and heterogeneity of HCC (135). Lentivirus-mediated overexpression or CRISPR/Cas9 knockout experiments have shown that glutaminase 1 (GLS1) regulates stemness and serves as a therapeutic target for the elimination of CSCs by inhibiting reactive oxygen species (ROS)/Wnt/β-catenin signaling *in vitro* and *in vivo*, while *GLS1* knockout inhibits tumorigenicity *in vivo* (136). Protein kinases play a key evolutionary conserved role in Wnt/β-catenin signaling and have been widely discussed as one of the most important drug targets in HCC therapy (34). However, inhibition of Wnt/β-catenin signaling alone is unlikely to significantly improve the prognosis of patients with HCC, and future research should focus on the combination of other therapies to improve the efficacy of Wnt/β-catenin signaling inhibitors. Currently, anti-HCC compounds targeting the inhibition of Wnt/β-catenin signaling are the most widely researched, most of which are in the clinical and preclinical research stages. As more research data become available, achieving the best personalized treatment for HCC in the future represents the ideal goal (137–140). Inhibition of Wnt/β-catenin signaling reduces the activation, proliferation, migration, and progression of HCC cells, which has attracted extensive attention from many researchers worldwide. At present, several institutions are developing targeted drugs.

### 2.6 JAK2/STAT3 signaling

Many studies suggest that Janus kinase2 (JAK2)/STAT3 signaling is closely related to hepatic fibrosis, and HSC activation is a key factor in the progression of hepatic fibrosis (39). Following the occurrence of homologous or heterooligomerization of specific receptor subunits on the HSC membrane with platelet-derived growth factor, leptin, TGF-β, and other cytokines promoting hepatic fibrosis, the receptor-coupled JAKs are activated. The Tyr of the cytoplasmic segment of the receptor is then phosphorylated to the protein anchor site containing SH2, and downstream signaling protein molecules are activated to enable the target genes to play a role. Tyr residues (Tyr705) and Serine (Ser) residues (Ser727) at the end of the STAT protein can also be phosphorylated by JAKs to activate the STAT protein and form homologous or heterologous STAT protein dimers into the nucleus to bind the promoter of corresponding target genes, thus activating target gene transcription of HSCs and participating in the development of hepatic fibrosis (38). The results of animal experiments have shown that p-JAK2/JAK2 and p-STAT3/STAT3 protein levels were significantly higher in the fibrosis model group compared with those in the control group, suggesting that JAK2/STAT3 signaling was activated in the liver tissues of rats during dimethylnitrosamine-induced hepatic fibrosis and was involved in the development of hepatic fibrosis (40) (**Figure 2**). Moreover, many studies have shown that JAK2/STAT3 signaling activation is involved in the occurrence and development of hepatic fibrosis and HCC (**Table 1**).

#### 2.6.1 JAK2/STAT3 signaling and hepatic fibrosis

JAK2/STAT3 signaling is a research hotspot in fibrotic disease, but the role of JAK2/STAT3 in the progression of hepatic fibrosis remains controversial (37, 141). Ding et al. found that HCC is closely related to chronic inflammation and fibrosis, which is called the inflammation–fibrosis–cancer axis (142). In addition, compared with the control group, JAK2/STAT3 signaling was significantly upregulated in the DEN exposure group, in that 100% of the rats in the DEN exposure group developed liver tumors at 20 weeks, with accompanying inflammatory and fibrotic stages correlated with exposure time. Most literature studies have reported that inhibiting JAK2/STAT3 signaling of HSC alleviates hepatic fibrosis both *in vivo* and *in vitro* (38, 39). Yang et al. found that magnesium (Mg) isoglycyrrhizinate improved high-fructose-induced liver fibrosis in rats by inhibiting JAK2/STAT3 and TGF-β1/Smad signaling by increasing microRNA-375-3p, but the effect of a direct interaction between JAK2/STAT3 and TGF-β1/Smads signaling on hepatic fibrosis *in vivo* needs further study (143). Studies have confirmed that quiescent HSCs, hepatocytes, and bile duct cells with an epithelial phenotype can be transformed into fibroblasts with a mesenchymal phenotype through EMT and participate in the occurrence and development of hepatic fibrosis (40). Xu et al. reported that the natural extract of brown algae, named propylene glycol alginate sodium sulfate (PSS), could significantly prevent liver injury and fibrosis induced by BDL and CCl_4_ in mice. Mechanistically, PSS significantly inhibits the activation of HSCs in LX-2 cell lines incubated with 10 ng/mL TGF-β1 for 24 h by inhibiting the anti-autophagy effect of JAK2/STAT3 signaling (144). In conclusion, STAT3 may play a dual role in hepatic fibrosis because of different upstream regulatory factors. Currently, most studies suggest that STAT3 mainly plays a role in promoting hepatic fibrosis, and its expression level may be positively correlated with the degree of fibrosis.

#### 2.6.2 JAK2/STAT3 signaling and HCC

STAT3 is closely related to the development and prognosis of HCC and can be adjusted by different target genes, affecting cell proliferation, apoptosis, invasion, migration, angiogenesis, and immune escape. Many studies have reported that directly inducing apoptosis of HepG2 cells by downregulating the JAK2/STAT3 signal transduction pathway may contribute to the development of new therapeutic strategies for HCC (145). The humanized-immune-system HCC mouse model is a newly developed animal model for the study of new targets of HCC immunotherapy, which has wide application prospects (146). Zhao et al. first demonstrated that intratumor human cluster of differentiation–positive (hCD14+) cells could produce IL-33 through DAMP/TLR4/activator protein 1, which increased IL-6 in other intratumor immune cells and activated JAK2/STAT3 signaling in HCC (146). However, the mechanism of crosstalk between human HCC and the human immune system still needs to be studied in depth (41, 147). Wei et al. recently found that TAMs mediate the EMT program of tumor cells before the invasion and speculated that EMT-programmed tumor cells can in turn recruit macrophages. In addition, crosstalk exists between TAMs and cancer cells in the TME, and IL-6 secreted by TAMs binds to the IL-6 receptor on the surface of cancer cells and phosphorylates STAT3 (pSTAT3) (148). It has been widely verified that abnormal activation (phosphorylation) of JAK/STAT3 signaling upregulates EMT, promoting HCC progression (42, 149). Liu et al. found that the homologous to the E6-AP carboxy terminus domain and RCC1-like domain 2 (HERC2) promote inflammation-induced stemness and immune evasion in HCC cells through JAK2/STAT3 signaling, the underlying mechanism of which is related to the regulatory effect of HERC2 on JAK2/STAT3 signaling participating in the crosstalk between cancer stemness and immune evasion (150). Cytokine activation of STAT3 is mediated by Tyr phosphorylation of JAK, and crosstalk between JAK/STAT3 signaling activation and EMT-mediated metastasis has been observed in HCC over the past decade (43–45). JAK2/STAT3 signaling is involved in many biological processes, including cell proliferation, angiogenesis, and migration in HCC (46).

### 2.7 CTGF/integrin/FAK signaling

CTGF is a multifunctional secretory polypeptide that regulates biological activities such as cell proliferation, differentiation, and adhesion and plays an important role in tissue damage repair and ECM synthesis (151). Integrins are receptor proteins on the surface of HSCs. Recent studies have confirmed that CTGF plays a positive regulatory role in the development of hepatic fibrosis (152). Focal adhesion kinase (FAK) is an important cytokine in integrin signaling, and activation of FAK further initiates a series of downstream cell signaling cascades (**Figure 3**). Currently, many studies have found that CTGF/integrin/FAK signaling is associated with hepatic fibrosis and HCC (47, 48, 153) (**Table 1**).

#### 2.7.1 CTGF/integrin/FAK signaling and hepatic fibrosis

FAK is an important cytokine in the integrin signaling system. After the integrin binds to the extracellular ligand molecule, the cytoplasmic end binds to the N end of the FAK protein to transmit extracellular signals (**Figure 3**). FAK is activated by phosphorylation, which further activates a series of downstream protein kinases (152). Currently, invasive biopsy is still the gold standard for the diagnosis of hepatocellular fibrosis. Shao et al. developed a new [18F]-Alfatide imaging targeting integrin αvβ3, which provides a noninvasive method for evaluating the expression and function of integrin αvβ3 on activated HSCs in the injured liver (47). Researchers have successfully quantitatively evaluated the level of liver integrin αvβ3 and the progression of hepatic fibrosis in mouse models induced by CCl_4_ and BDL; however, these studies are only at the preclinical stage, and further studies are needed (48, 153, 154). HSC activation and MYF differentiation are central to hepatic fibrosis and occur during ECM deposition (49, 155, 156). Integrin has been extensively investigated as a drug target for hepatic fibrosis, and a variety of antifibrotic drugs targeting integrin have entered the clinical research stage (50, 51, 157). Currently, clinically used integrin inhibitors, such as abciximab (targeting αIIbβ3 on platelets), natalizumab (targeting α4β1 on T-cells), vedolizumab (targeting α4β7 on T-cells), and lifitegrast (targeting αLβ2 on T cells), usually bind specifically to their target (52). Shi et al. found that the promoter methylation of the *CTGF* underwent phenotypic changes in HSCs, becoming MYF-like cells expressing α-SMA. CTGF promoted phenotypic changes of HSCs into MYFs, whereas inhibition of CTGF promoter methylation enhanced this process, suggesting that CTGF group promoter methylation may negatively regulate hepatic fibrosis (158). *In vivo* experiments showed that the severity of hepatic fibrosis in a CCl_4_-induced rat liver fibrosis model was negatively correlated with the promoter methylation level of the *CTGF* in HSCs, suggesting that promoter methylation of the *CTGF* may prevent the occurrence of hepatic fibrosis. Therefore, low levels of promoter methylation of the *CTGF* may be a predisposing factor for the occurrence of hepatic fibrosis (158). After transfection of the *FAK* shRNA recombinant plasmid into HSCs, the migration and adhesion activities of the recombinant HSCs were significantly decreased compared with those of the control HSCs. In addition, *FAK* mRNA expression was significantly increased in acetaldehyde-activated HSCs, suggesting that FAK plays an important role in the activation of HSCs in hepatic fibrosis.

#### 2.7.2 CTGF/integrin/FAK signaling and HCC

HCC usually develops from hepatic fibrosis. CTGF has been found to be overexpressed in 93 human HCC tissues compared with human non-HCC tissues—mainly in HCC cells—and increased CTGF expression was associated with the clinicopathological malignancy of HCC (159). A previous study confirmed that tumor cell-derived CTGF is a building block in the HCC microenvironment, activating nearby HSCs and delivering growth-promoting signals to HCC cells (159). This interaction is easily suppressed by anti-CTGF antibodies, suggesting that pro-tumor crosstalk between HCC cells and HSCs provides an opportunity for therapeutic intervention in HCC. Li et al. found that activation of the integrin β1/Piezo1 contributes to matrix stiffness-induced angiogenesis in HCC, and that high Piezo1 expression is predictive of poor prognosis (160). Integrins are key players in the spread of tumor cells and are expressed at different levels at different stages of tumor development (161, 162). Sarker et al. have extensively summarized the effects of integrin crosstalk with growth factor receptors on growth factor signaling and reviewed the evidence supporting the mechanistic regulation of integrin crosstalk with growth factor signaling, which has important implications for normal cell physiology and anticancer therapy (163). A new study has observed that enhanced radiation-induced G2/M cell cycle arrest is dependent on crosstalk between integrinβ1 and growth factor receptor signaling, which is a new research direction to elucidate the signaling underlying the EGFR/integrinβ1 crosstalk, which may support the development of advanced molecular targeted therapies in radiation oncology (164). Integrin crosstalk refers to the mechanism by which changes in the expression of one integrin subunit or activation of an integrin heterodimer may interfere with the expression and/or activation of other integrin subunits in the same cell. This phenomenon was first described in K562 erythroleukemia cells in 1994 (53, 165, 166). Samaržija et al. reviewed the evidence for integrin crosstalk in a range of cellular systems, with a particular emphasis on cancer, and described the molecular mechanisms of integrin crosstalk, the influence of cell fate determination, and the contribution of crosstalk to therapeutic outcomes (53). As a key player in many cancer features, integrins have been recognized as valuable tumor therapeutic targets (167, 168). Gahmberg et al. conducted a detailed summary of the regulation of integrin-integrin crosstalk on dynamic cell adhesion and found that although the mechanisms leading to integrin crosstalk are incompletely understood, they usually involve intracellular signaling and are also used by other cell surface receptors (169). The phosphorylation of integrins and key intracellular molecules play a crucial role in integrin-cytoplasmic interactions, which, in turn, influence integrin activity and crosstalk, with the integrin β-chain playing a central role in regulating crosstalk. In addition to integrin-integrin crosstalk, crosstalk can occur between integrins and related receptors, including other adhesion and growth factor receptors.

### 2.8 PI3K/AKT signaling pathway

PI3K/AKT signaling plays a pivotal role in intracellular and extracellular signal transduction and is also involved in cell proliferation, apoptosis, invasion, and metastasis. PI3K is a crucial protein molecule involved in PI3K/AKT signaling (170). Activated PI3K can promote changes in the downstream substrate AKT and also activate the translocation of *AKT* to the nucleus, thus causing the expression of downstream-related genes and regulating cell proliferation, metabolism, and a series of physiological functions (**Figure 3**). Mammalian target of rapamycin is the downstream molecule of AKT and is normally activated to play an important role in the regulation of the cell cycle, cell growth, and proliferation (171). The mechanism of action of dexmedetomidine, apatinib, rosiglitazone, sorafenib, metformin, and baicalin may be downregulating the expression of PI3K and phosphorylation of AKT, while activating the apoptotic signaling of caspases to promote apoptosis. PI3K/AKT signaling is activated in 30%–50% of patients with HCC, and upregulation of phosphorylated AKT (p-AKT) is associated with poor survival and tumor vascular invasion in patients with HCC (172) (**Table 1**). PI3K/AKT signaling is also associated with the inhibition of HCC apoptosis. Therefore, PI3K/AKT signaling may be key to HCC drug development.

#### 2.8.1 PI3K/AKT signaling and hepatic fibrosis

The PI3K/AKT signaling pathway is involved in many processes that regulate HSC activation, including collagen synthesis and cell proliferation. HSC activation is a key step in hepatic fibrosis that requires global reprogramming of gene expression, which is regulated by multiple mechanisms, including epigenetic regulation, such as DNA methylation (173, 174). Studies have confirmed that phosphoenolpyruvate carboxykinase 1 deficiency promotes platelet-derived growth factor AA expression through PI3K/AKT signaling and activates HSCs via hepatocyte-HSC crosstalk, and that this important crosstalk between hepatocytes and HSCs is mediated by paracrine signaling (54, 55). However, the effect of activating or inhibiting PI3K/AKT signaling on hepatic fibrosis remains controversial (64, 175, 176). Recent studies have shown that TGF-β crosstalk with PI3K/AKT signaling occurs in a Smad-independent manner, suggesting that specific crosstalk between macrophages and HSCs via soluble proteins could be an area for further study (177). Damaged hepatocytes and activated immune cells convert quiescent HSCs into MYFs through crosstalk. Recently, it has been reported that DC function is associated with PI3K/AKT signaling, involving functional reprogramming of immune cells, control of cellular responses, and regulation of hepatic fibrosis (56). PI3K/AKT signaling is involved in reprogramming the activity of many immune cells, particularly DCs and macrophages. The latest study by Xiang et al. showed that kinsenoside treatment improved the hepatic inflammatory microenvironment of hepatic fibrosis and reprogrammed intracellular glycolysis by inhibiting the migration and maturation of DCs via inhibition of PI3K/AKT signaling (56). PI3K/AKT signaling is an important pathway regulating HSC proliferation and transdifferentiation in the liver (57, 58). Further investigation revealed that hepatic fibrosis involved complex mutual crosstalk between PI3K/AKT signaling and TGF-β1/Smads signaling, and further study is required (59, 65).

#### 2.8.2 PI3K/AKT signaling and HCC

PI3K can also inhibit tumor cell apoptosis, enhance cell resistance to chemotherapy-induced apoptosis, and promote postoperative tumor growth (60, 178, 179). Crosstalk between stromal and HCC cells changes the characteristics of HCC cells and promotes their growth and metastasis. FGF is also known to play an important role in the development of HCC. Indeed, Liu et al. have demonstrated that matrix stiffness contributes to the mechanical signal transduction of HSCs and promotes the growth and metastasis of HCC cells through the secretion of FGF2 (108). Li et al. also reported for the first time that activated HSC-derived COMP regulates the gene expression of mesenchymal and matrix metalloproteinase (MMP) in HCC cells through CD36, causing abnormal phosphorylation of ERK and AKT, with crosstalk between PI3K/AKT and MEK/ERK signaling (106). Intriguingly, zebrafish share the same molecular pathways as humans. Experiments using a zebrafish HCC model found that aloperine reduced the proliferation of Huh7 cells in a dose- and time-dependent manner, suggesting that the PI3K/AKT cell cycle is an important central node against HCC (61, 180, 181). PI3K/AKT signaling is responsible for metabolic reprogramming and glycolytic induction in HCC and is therefore a promising therapeutic target. Targeting PI3K/AKT signaling has a positive biological impact and a very promising therapeutic prospect in the prevention of HCC (62). Many studies have reported that PI3K/AKT is widely expressed in various types of cancer cells, representing a promising target for tumor therapy (63, 182–185). Metabolic reprogramming is a novel feature of cancer that involves multiple effects and steps and has been recognized as a hallmark of cancer (186, 187). Moreover, the reprogramming of gene expression during EMT and non-transcriptional changes are triggered and regulated by signaling in response to extracellular signals (188). Activation of PI3K/AKT signaling promotes glucose uptake and glycolysis, increases tumor cell proliferation, inhibits apoptosis and autophagy, and promotes cell survival (189–191). During HCC metastasis, PI3K/AKT stimulates EMT and increases MMP expression. Anticancer drugs regulate the proliferation, stemness, and apoptosis of HCC cells by targeting PI3K/AKT signaling (**Table 1**). Future studies translate these findings to increase and realize more effective treatment options for patients with HCC.

### 2.9 Ferroptosis

The term ferroptosis was coined in 2012 because erastin and RAS selective lethal small molecules (RSLs) selectively induce non-apoptotic programmed cell death in various cancer cells, which can be blocked by iron chelating agents (192) (**Figure 3**, **Table 1**). Ferroptosis is an iron-dependent form of programmed cell death characterized by the accumulation of large amounts of lipid peroxidation in cells and an imbalance in the Redox state (66, 68, 69). Ferroptosis is associated with various signaling pathways, including amino acid and iron metabolism, ferritin autophagy, and cell adhesion (193–195). Many studies have shown that ferroptosis is closely related to HCC and hepatic fibrosis (70–73, 196). Although hepatic fibrosis has consistently been a global health problem, there remains a lack of effective treatment. The discovery of ferroptosis provides a new perspective to solve this problem.

#### 2.9.1 Ferroptosis and hepatic fibrosis

Ferroptosis is a new form of regulatory necrosis involved in various hepatic diseases, including cancer, hepatic fibrosis, and ischemia–reperfusion injury (66, 192). *In vitro* assays showed that treatment with artesunate significantly triggered ferroptosis in activated HSCs (68). Yi et al. found that berberine alleviated hepatic fibrosis induced by TAA and CCl_4_ in mice by inducing ferrous redox activation of ROS-mediated HSC ferroptosis, the mechanism of which involves ubiquitination-related ubiquitin–proteasome pathway-autophagy crosstalk in HSCs (69). However, the opposite view persists. The reason for these contradictory results may be related to the insufficient research on the relationship between ferroptosis and hepatic fibrosis (193–195). Ferroptosis is the result of iron homeostasis, iron deficiency, and acquired and inherited iron overload, resulting in iron metabolism disorders (70, 196). Ferroptosis has unique biological energy and morphological characteristics, which are different from other forms of programed cell death. However, the specific mechanism of ferroptosis in the process of hepatic fibrosis is still unclear, and some new experimental results are controversial (71–73). Although ferroptosis and apoptosis involve different mechanisms, a potential link between ferroptosis and apoptosis in HSCs still needs to be studied. Future research will focus on whether ferroptosis and apoptosis are closely related and whether crosstalk exists between them (73, 197, 198). In summary, the aforementioned studies suggest that inducing ferroptosis in HSCs may be a viable strategy for the treatment and/or prevention of hepatic fibrosis (67, 74, 199). However, challenges remain regarding the ability to selectively induce HSC ferroptosis with minimal impact on other healthy liver cells. Future studies are needed to determine the exact molecular mechanisms of ferroptosis and lipid metabolism involved in the pathogenesis of hepatic fibrosis (200, 201).

#### 2.9.2 Ferroptosis and HCC

Increasing evidence supports the idea that activating ferroptosis may effectively inhibit HCC cell growth, thus providing a scientific basis for targeting ferroptosis as a new therapeutic strategy for HCC (202, 203). Thus, ferroptosis-inducing compounds are widely considered a promising approach for the development of novel anticancer drugs (204–207) (**Table 1**). Indeed, since its discovery in 2021, multiple studies have found that ferroptosis is the dominant pattern of radiation-induced cell death in HCC (75, 76, 208). Moreover, recent studies have reported direct crosstalk between ferroptosis and anti-tumor immunity, showing that tumor-infiltrating lymphocyte-mediated ferroptosis can effectively enhance the efficacy of immune checkpoint inhibitors (209–211). Xu and colleagues conducted an in-depth study on the prognostic characteristics of lncRNA associated with ferroptosis and established a promising prognostic model for HCC based on the differentially expressed lncRNA associated with ferroptosis, which can be used for prognostic prediction and selection of patients for immunotherapy. This is the first report on the effects of ferroptosis-related lncRNAs on the prognosis and immune response of the HCC population, providing a promising strategy to guide individualized therapy and improve prognosis prediction (210). Here, we summarized the typical involvement of ferroptosis in hepatic fibrosis and the HCC process and discussed the potential mechanism. Many studies have found that inducing ferroptosis inhibits hepatic fibrosis and HCC progression (212, 213). Preclinical studies of many natural products, chemicals, and drug carriers have revealed their ability to trigger ferroptosis; indeed, ferroptosis inducers are considered a key development direction in the future, with high efficiency and low toxicity.

### 2.10 YAP/HIF-1α signaling

YAP is an important transcriptional coactivator and a key nucleoplasm shuttle protein that is abnormally highly expressed in many malignant tumors, including HCC, and plays a role in transcriptional regulation mainly in the nucleus. Hypoxia-inducible factor-1α (HIF-1α) is a transcriptionally active nucleoprotein, the regulation of which through cell signaling was first discovered by Semenza and colleagues in 1995. Over the past few decades, a large body of evidence has shown that HIF-1α is closely associated with hepatic fibrosis (214). HIF-1α is stably expressed during hypoxia and forms a dimer with HIF-1β, inducing downstream gene transcription and hypoxia response (215–217). HIF-1α regulates VEGF under hypoxic conditions, controls angiogenesis, cell proliferation, and metastasis, and plays an important role in the occurrence and development of hepatic fibrosis and HCC (77, 78, 218, 219). In tumor cells, YAP and HIF-1α can bind to each other and stabilize HIF-1α proteins in the nucleus, forming the YAP-HIF-1α complex. Hypoxia regulates YAP phosphorylation and promotes *YAP* nuclear translocation, allowing it to bind to HIF-1α and promote its transcriptional activity (220–223) (**Figure 3**). The role of the YAP protein in the metabolic reprogramming of tumor cells has received increasing attention by researchers and has become a current research focus.

#### 2.10.1 YAP/HIF-1α signaling and hepatic fibrosis

Hypoxia is an important feature of hepatic fibrosis, which can upregulate the expression of HIF-1α, promote the activation of HSCs, and lead to hepatic fibrosis. Improving the anoxic environment at the site of hepatic fibrosis and downregulating the expression of HIF-1α to the maximum extent can improve the therapeutic effect of hepatic fibrosis (215, 216). HIF-1α is known to stimulate collagen synthesis and chemotaxis in HSCs, as well as induce HSC migration, with activated HSCs known to play a dominant role in sinusoidal structural changes during fibrosis through crosstalk with LSECs. HIF-1α also regulates HSC metabolic reprogramming through the deactivation of glycolytic genes (216). However, controversial conclusions still exist (217). Indeed, Fan et al. recently found that HIF-1α signaling plays an important role in the metabolic reprogramming of LPS-activated macrophages from glycolysis to oxidative phosphorylation induced by Celastrol (218). Multiple studies have confirmed that inflammation is the driving force behind hepatic fibrosis (77, 217, 218). As a transcription factor, HIF-1α reprograms the adaptive response of hypoxic metabolism and can induce fibrosis in multiple tissues (78). In addition, in tumor and stem cells, succinate induces HIF-1α to reprogram energy metabolism under hypoxic conditions. Intracellular succinate accumulation activates HSCs in a receptor-independent manner, while induction of HIF-1α acts as a transcription factor to reprogram cellular metabolism in response to stress (78, 219–221). HIF-1α represents an important transcription factor that promotes hepatic fibrosis, making it a potential therapeutic target (222–224). Under hypoxic conditions, HIF-1α subunits are stabilized by inhibition of proline hydroxylase and thus accumulate in the nucleus. *HIF-1* binds to hypoxic response elements and regulates the transcription of hundreds of genes involved in multiple processes, including angiogenesis, cell proliferation, erythropoiesis, metabolic reprogramming, and apoptosis/survival, in response to cell damage caused by hypoxia (224–226). Many studies have shown that HIF-1α mediates oxygen homeostasis, regulates the expression of multiple hypoxia stress protein genes, and significantly increases the expression in hepatic fibrosis tissues (79–81, 227, 228) (**Table 1**). Sun et al. found that microRNA-21 alleviates the abnormal crosstalk of hepatocytes and HSCs by inhibiting HIF-1α/VEGF signaling, ameliorating arsenite-induced hepatic fibrosis (229). Both microRNA-98 and miR-345–5p ameliorate hepatic fibrosis through inhibiting HIF-1α signaling (230, 231). However, microRNA-322/424 promotes angiogenesis to aggravate hepatic fibrosis by activating the HIF-1α signaling (232). Hypoxia significantly increases the levels of proinflammatory and profibrotic factors, and inhibition of HIF-1α is key to reducing adipose fibrosis and inflammation (82). Zhang et al. reported that Oroxylin A alleviated the angiogenesis of LSECs in hepatic fibrosis by suppressing YAP/HIF-1α signaling (83). Moreover, *in vitro* interference with YAP was found to significantly down-regulate HIF-1α protein expression, whereas the YAP plasmid showed the opposite effect in cellular assays (233–235). Recently, it has been shown that overexpression of HIF-1α-antisense RNA 1 inhibits the progression of hepatic fibrosis (236, 237).

#### 2.10.2 YAP/HIF-1α signaling and HCC

In general, HCC occurs primarily in the context of cirrhosis resulting from chronic inflammation and advanced fibrosis. Extensive hepatic fibrosis is observed in the paracancer tissues of patients with HCC. Previous studies have shown that hypoxia can promote the release of fibrotic mediators (84). HCC cells can reprogram their energy metabolism to aerobic glycolysis, a phenomenon known as the Warburg effect. HCC is often associated with intratumor hypoxia due to the high oxygen metabolism of rapidly proliferating tumor cells. This hypoxic microenvironment promotes tumor aggressiveness and treatment resistance by activating the HIF regulatory pathway (238). Existing *in vivo* and *in vitro* evidence supports that increased matrix stiffness enhances the malignant characteristics of HCC cells and promotes invasion and metastasis in various ways, including by triggering the occurrence of EMT, promoting the formation of a pre-metastatic niche, enhancing stemness characteristics, upregulating the expression of invasion/metastasis-related genes, affecting the reprogramming of glucose and lipid metabolism, and reducing the efficacy of chemotherapy (160). Hypoxia is a key factor in inducing the transcription of HIF-1α encoding *HIF1A* and the accumulation of HIF-1α protein to promote angiogenesis (85, 239–241). HIF-1α is a key regulator of glycolytic metabolism, which mediates energy metabolic reprogramming in HCC (242). Inhibiting or interfering with the expression of HIF-1α effectively restrains energy metabolism and growth in HCC glycolytic metabolism. HCC cells undergo metabolic reprogramming, which is essential for subsequent rapid tumor growth, with lipid metabolism reprogramming identified as one of the new hallmarks of cancer (243). In the liver tissues of patients with HCC, both fatty acid binding protein 5 (FABP5) and HIF-1α are up-regulated, and their protein expression levels are associated with poor prognosis. Seo et al. recently reported that fatty acid-induced FABP5 upregulation drives HCC progression through HIF-1α-driven lipid metabolic reprogramming (243). Yang et al. recently found that hepatitis B virus X-interacting protein drives the metabolic reprogramming of HCC cells through methyltransferase-like protein 3-mediated m6A modification of HIF-1α (244). In addition, inhibition of HIF-1α and HIF-2α activity interferes with tumor growth and vascularization as well as reprogramming of the tumor immune microenvironment to promote anti-tumor immunity and improve the response to anti-programmed cell death protein 1 therapy (86, 245–248). The role of hypoxia-induced HIF-1α in HCC progression has been extensively studied, and it has been confirmed that hypoxia stress in HCC cells promotes the binding of YAP to HIF-1α in the nucleus (249–251). Bao et al. conducted a detailed review of the role of HIF-mediated metabolic reprogramming in HCC drug resistance and found that HIF-induced glucose metabolism, glucose uptake, and glycolysis were activated in HCC cells under hypoxic conditions, which promoted glucose uptake and satisfied the glucose requirement for the growth of hypoxic cancer cells (252). Moreover, long-chain non-coding RNA expressed in HCC tissues was increased by activating HIF-1α signaling to enhance HCC cell activation and proliferation, thereby promoting HCC progression (253–256). Overexpression and activation of YAP are associated with poor prognosis in patients with HCC, possibly because it promotes tumor progression and/or metastasis. Previous research has found that the existence of nuclear *YAP* is possible due to its combination with DNA transcription factors, which helps maintain its activity to help stabilize HIF-1α. Dai and colleagues showed that YAP signaling activation is not dependent on HIF-1α under the condition of low oxygen (257–259). A high density of tumor-infiltrating plasmacytoid DCs (pDCs) is associated with poor prognosis in patients with HCC. Hypoxia-induced extracellular adenosine (eADO) significantly enhances pDC recruitment to tumors via adenosine A1 receptors (ADORA1). Mechanistically, *HIF-1α* transcriptionally upregulated the expression of exonucleogenases CD39 and CD73, which are essential for eADO production. Targeted recruitment of pDCs may become potential auxiliary HCC immunotherapy strategies, with crosstalk between inflammation and immunity reported previously (260). Reprogramming of lipid metabolism induced by HIF-1α has also been identified as a hallmark of cancer (261). Hypoxia increases the proportion of HCC cells with stem cell characteristics, whereas specific small ubiquitin-like modifier protease 1 (SENP1) promotes hypoxia-induced HCC cell stemness through HIF-1α oxygenation and a SENP1/HIF-1α positive feedback loop. Drugs specifically targeted to inhibit SENP1 may provide a novel therapeutic approach for HCC (262–264). It is known that there is a significant correlation between the anoxic microenvironment and sorafenib resistance. A new study found that β2 adrenergic receptor signaling negatively regulates autophagy, promotes HIF-1α stabilization, and reprograms glucose metabolism in HCC cells, leading to sorafenib resistance (265–267). YAP is highly expressed in the liver tissues of patients with HCC and is positively correlated with HIF-1α and pyruvate kinase isoform M2 (PKM2) expression. Gene set enrichment analysis has revealed that *YAP* overexpression in the liver tissues of patients with HCC under hypoxic conditions is closely correlated with *HIF-1α* (268). Hypoxia promotes glycolysis of HCC cells by reducing *p-YAP* expression and triggering *YAP* nuclear translocation. *YAP* activation directly interacts with *HIF-1α* in the nucleus, maintaining *HIF-1α* stability to activate *PKM2* transcription. YAP/HIF-1α signaling regulates HCC glycolysis and may represent a novel therapeutic target for HCC. However, whether YAP reprograms HCC cell metabolism requires further study.

## 3 Conclusion

The characteristics of fibrotic signaling that are common to cancer can be summarized as follows: 1) After reviewing the literature, we found ten fibrotic signals in HCC, all of which are related to inflammation, suggesting that inflammation plays a central role in the development of HCC. 2) When cells are stimulated by different factors, cell signals are transmitted from the extracellular to the intracellular space, and finally enter the nucleus to perform different physiological functions. In addition, fibrotic signals in HCC mediate HCC progression through critical regulation of various immune cells in the immune microenvironment in the liver to maintain a balance between immune tolerance and activation. 3) By activating or inhibiting these 10 fibrotic signals in HCC, different drugs inhibit the activation, proliferation, migration, invasion, and apoptosis of HSCs, CAFs, and HCC cells (**Figures 2**, **3**, **Table 1**). 4) The inhibition of metabolic reprogramming of HSCs or HCC cells by different drugs targeting various fibrotic signaling pathways in HCC is a focus of current research. 5) Crosstalk between HSCs/MYFs and HCC cells in the microenvironment alters the properties and facilitates the growth, proliferation, migration, and invasion of HCC cells. 6) Anti-HCC drugs regulate the stemness of HCC cells by targeting various fibrotic signals in HCC, which is also a current research focus. HCC can be distinguished from other cancers in terms of fibrotic signaling as follows: 1) The precancerous fibrotic microenvironment of chronic liver disease is characterized by neovascularization, inflammation, and fibrosis. Fibrotic signals of HCC are more closely related to inflammation than those of other cancers. 2) Inflammatory fibrosis of the liver leads to immune tolerance to the microenvironment, which prevents the immune system from recognizing and clearing malignant transformed hepatocytes, MYFs, and CAFs. As the liver contains several types of immune cells, the fibrotic signal of HCC is more closely related to immunity than that of other cancers. 3) The liver is the largest metabolic organ in the body, and it is currently a hot research topic, in which studies have focused on inhibiting the metabolic reprogramming of HCC cells and the formation of HIF. 4) The fibrosis signals of HCC involve multiple cells, and the crosstalk between different cells and signaling pathways is more complex and requires in-depth research. Finally, the occurrence and development of HCC can be inhibited to varying degrees by regulating the above eight fibrosis signaling pathways, many of which interact with each other. New drugs for the prevention and treatment of HCC targeting different fibrosis signaling pathways continue to appear, and those with good targeting and minimal side effects will likely be developed in the future (**Table 1**).

## Author contributions

Writing of the manuscript: LS, WX, and DZ. Developing the idea for the article and critically revising it: FW and JL. Supervision: XL. All authors contributed to the article and approved the submitted version.
